# Mechanism of Pydiflumetofen Resistance in *Fusarium graminearum* in China

**DOI:** 10.3390/jof9010062

**Published:** 2022-12-30

**Authors:** Feng Zhou, Huan-Huan Zhou, Ao-Hui Han, Kou-Yun Guo, Tian-Cheng Liu, Yan-Bing Wu, Hai-Yan Hu, Cheng-Wei Li

**Affiliations:** 1 Henan Engineering Research Center of Crop Genome Editing/Henan International Joint Laboratory of Plant Genetic Improvement and Soil Remediation, Henan Institute of Science and Technology, Xinxiang 453003, China; 2 Postdoctoral Research Base, Henan Institute of Science and Technology, Xinxiang 453003, China; 3 School of Food Science and Engineering, Henan University of Technology, Zhengzhou 450001, China; 4 Henan Engineering Research Center of Green Pesticide Creation and Pesticide Residue Monitoring by Intelligent Sensor, Henan Institute of Science and Technology, Xinxiang 453003, China

**Keywords:** *Fusarium graminearum*, pydiflumetofen, fungicide resistance, molecular mechanism, cross-resistance

## Abstract

Fusarium head blight (FHB), which is primarily caused by *Fusarium graminearum*, is a widespread and devastating disease of wheat. In the absence of resistant varieties, the control of FHB relies heavily on the application of fungicides, and the new generation SDHI fungicide, pydiflumetofen, has recently been registered in China for the control of FHB in wheat. The current study explored three genetically stable, highly resistant laboratory mutants (S2-4-2R, S27-3R, and S28-2R, with EC_50_ values of 25.10, 28.57, and 19.22 μg/mL, respectively) to investigate the potential risks associated with pydiflumetofen resistance. Although the mycelial growth of the mutants differed little compared to their parental isolates, the study found that the resistant mutants exhibited significantly reduced (*p* < 0.05) levels of sporulation and pathogenicity, which suggests a significant fitness cost associated with pydiflumetofen resistance in *F. graminearum*. Sequence analysis of the Sdh target protein identified numerous amino acid substitutions in the predicted sequences of the four subunits: FgSdhA, FgSdhB, FgSdhC, and FgSdhD. Indeed, the mutants were found to have a series of substitution in multiple subunits such that all three exhibited five identical changes, including Y182F in the FgSdhA subunit; H53Q, C90S, and A94V in FgSdhB; and S31F in FgSdhC. In addition, gene expression analysis revealed that all of the *FgSdh* genes had significantly altered expression (*p <* 0.05), particularly *FgSdhA* and *FgdhC*, which exhibited remarkably low levels of expression. However, the study found no evidence of cross-resistance between pydiflumetofen and tebuconazole, fludioxonil, prochloraz, fluazinam, carbendazim, pyraclostrobin, or difenoconazole, which indicates that these fungicides, either in rotation or combination with pydiflumetofen, could mitigate the risk of resistance emerging and provide ongoing control of FHB to ensure high and stable wheat yields.

## 1. Introduction

Wheat (*Triticum aestivum* L.) is a critical grain crop that provides the staple food for approximately 40% of the global population [1], and ensuring sustainable wheat production is therefore crucial to world food security. In 2020, China was the leading producer of wheat, with an annual yield in excess of 130 million tons, and yet was still the second largest importer, which illustrates the importance of maintaining good harvests to both sustain domestic consumption as well as to reduce pressure on global wheat markets (https://worldpopulationreview.com/country-rankings/wheat-production-by-country, accessed on 30 October 2022). However, the production of wheat in China is under constant threat from Fusarium head blight (FHB), which is a widespread and devastating disease [2]. The FHB complex, which is primarily caused by *Fusarium graminearum*, not only results in yield loss and smaller grains but can also dramatically affect grain quality as a consequence of contamination with harmful mycotoxins such as deoxynivalenol (DON), which pose a threat to both human and animal health [2,3]. In the absence of effective and stable wheat varieties with high resistance to *F. graminearum*, the application of fungicides remains the most effective strategy to control FHB [3]. However, unfortunately inappropriate and over use of such compounds for an extended period of time has already led to the emergence of resistance to some of the most effective fungicides, including carbendazim and tebuconazole, within field populations of *F. graminearum* in China [3,4,5]. It is therefore extremely important to continuously monitor for signs of resistance in order to adjust chemical control strategies accordingly, and the need for alternative novel compounds for the management of FHB is increasing in urgency.

Pydiflumetofen is a new-generation succinate dehydrogenase inhibitor (SDHI), which was recently developed by Syngenta Crop Protection and registered in China for the control of FHB in 2020 (http://www.chinapesticide.org.cn/hysj/index.jhtml, accessed on 30 October 2022). The mode of action of pydiflumetofen and other SDHI fungicides has been studied in detail, and it is generally accepted that they block the ubiquinone binding site of the succinate dehydrogenase (SDH) enzyme, also known as succinate ubiquinone oxidoreductase, which plays an essential role in the tricarboxylic cycle in the fungal mitochondria [6,7]. The SDH of *F. graminearum* consists of at least four nuclear-encoded subunits, including FgSdhA (FGSG_13136), FgSdhB (FGSG_05610), FgSdhC (FGSG_09012), and FgSdhD (FGSG_00743), which are all located in the mitochondria [8]. Although pydiflumetofen exhibits high activity against *F. graminearum* in artificial culture (Table 1), the observation from a recent study that the risk of developing resistance to SDHI fungicides should be categorized as medium to high is of great concern [9]. Previous research has shown that several amino acid changes in the sequences of the various Sdh subunits have been associated with resistance to SDHI fungicides in plant pathogenic fungi, including *Botrytis cinerea* and *Fusarium asiaticum* [10,11,12]. The main objectives of the current study were therefore to investigate the potential for pydiflumetofen resistance in *F. graminearum* and more specifically to (i) explore the fitness parameters of pydiflumetofen-resistant mutants of *F. graminearum*; (ii) identify the molecular basis of the observed resistance; and (iii) assess the potential for cross-resistance between pydiflumetofen and other commonly used fungicides.

## 2. Materials and Methods

### 2.1. Fungicides, Isolates, and Medium

Stock solutions of the technical-grade fungicides (Table S1) used in the current study were prepared in appropriate solvents and stored at 4 °C for no longer than 2 weeks before use. Pydiflumetofen, tebuconazole, fludioxonil, prochloraz, fluazinam, pyraclostrobin, and difenoconazole were dissolved in acetone, whilst the carbendazim was dissolved in 0.1 mol/L hydrochloric acid (HCl). Serial dilutions were freshly prepared for each experiment, and mycelial growth assays performed to confirm that the solvents had no effect on the growth of *F. graminearum* at the range of concentrations tested (data not shown).

The three wild-type *F. graminearum* isolates used in the current study, S2-4-2, S27-3, and S28-2, which were highly sensitive to pydiflumetofen, with EC_50_ values of 0.0235, 0.0486, and 0.2354 μg/mL, respectively, were originally collected from wheat ears exhibiting typical symptoms of FHB growing in the fields of China (Table 1). Repeated exposure to pydiflumetofen under laboratory conditions following the protocol of a previous study [13] resulted in the selection of three genetically stable mutants, S2-4-2R, S27-3R, and S28-2R, which were highly resistant to pydiflumetofen, with EC_50_ values of 25.10, 28.57, and 19.22 μg/mL, respectively (Table 1). Both the wild-type isolates and the resistant mutants were routinely maintained on potato dextrose agar (PDA: 20 g/L glucose, 200 g/L potato, and 20 g/L agar) at 24 °C.

### 2.2. Mycelial Growth, Sporulation, and Pathogenicity of Pydiflumetofen-Resistant Mutants of F. graminearum

The biological characteristics of the three resistant *F. graminearum* mutants (S2-4-2R, S27-3R, and S28-2R) were evaluated in comparison to their wild-type parental isolates (S2-4-2, S27-3, and S28-2), with their mycelial growth on PDA; sporulation in mung bean broth (MBB), which was prepared by boiling 40 g of mung bean seeds in one liter of distilled water for 30 min; and pathogenicity on wheat spikelets assessed according to the methods detailed in a previous study [13,14]. For mycelial growth assay, mycelial plugs (5 mm in diameter) of the test isolates were taken from the edge of 2-day-old colonies and transferred to fresh PDA plates that were then incubated at 24 °C with a 12 h photoperiod, and the resulting colonies were observed daily and the diameter of each measured at 24 h, 48 h, and 72 h post inoculation (hpi), and each isolate was represented by at least eight individual plates. For sporulation assay, the test isolates were initially established by transferring 5 mm mycelial plugs from 2-day-old PDA cultures to flasks containing 30 mL MBB. After 3 days of incubation at 24 °C with shaking (120 rpm), the resulting spores were harvested and counted using a hemocytometer (Shanghai Qiujing Biochemical Reagent Instrument Co., Ltd., Shanghai, China), and each isolate was represented by at least 3 flasks. Meanwhile, for the pathogenicity assay, colonies were initially established by transferring 5 mm mycelial plugs from 2-day-old PDA cultures to flasks containing 30 mL MBB. After 3 days incubation at 24 °C with 120 rpm shaking, the resulting spores were harvested and resuspended in serial dilutions of sterile water and counted using a hemocytometer. The pathogenicity of the six *F. graminearum* isolates was assessed using wheat ears grown under greenhouse conditions, and the test was conducted using intact wheat ears (Cultivar Bainong 307), which were inoculated with 10 μL of spore suspension (1 × 10^5^ spores/mL) at the beginning of the heading and flowering growth stage. Water-inoculated wheat ears served as negative controls. The severity of disease symptoms were evaluated for 14 days post inoculation (dpi) from the previous studies [14]. Each isolate was represented by at least 10 wheat spikelets.

### 2.3. Cloning and Sequencing of Four FgSdh Genes

Mycelium samples were prepared in accordance with the method of a previous study [13], and total genomic DNA extracted using the Omega bio-tek Fungal DNA Kit (Omega bio-tek Inc., Guangzhou, China) following the instructions of the manufacturer. The resulting DNA was used as a template for polymerase chain reaction (PCR) amplification of the four full-length *Sdh* genes, FgSdhA, FgSdhB, FgSdhC, and FgSdhD, using the following primer sets, which were designed with primer premier software (ver.6.0., PREMIER Biosoft, Palo Alto, CA, Canada): FgSdhA-F/FgSdhA-R, FgSdhB-F/FgSdhB-R, FgSdhC-F/FgSdhC-R, and FgSdhD-F/FgSdhD-R, respectively (Table S2). The PCR was performed using 50.0 μL reaction mixtures containing 25.0 μL 2×ES Taq Master Mix, 1.5 μL of template DNA, 2.0 μL each primer, and 21.5 μL ddH_2_O (CoWin Biosciences, Cambridge, MA, USA) and processed using a 96-well thermal cycler (Applied biosystems, Thermo Fisher Scientific, Waltham, MA, USA) with the following program: an initial denaturation at 95 °C for 2 min, following by 35 cycles of melting at 95 °C for 30 s, annealing at 58 °C for 120 s in the case of *FgSdhA* (57.5 °C for *FgSdhB*, 58 °C for *FgSdhC*, and 57 °C for *FgSdhD*), and extension at 72 °C for 120 s in the case of *FgSdhA* (50 s for *FgSdhB*, 40 s for *FgSdhC*, and 45 s for *FgSdhD*), with a final extension at 72 °C for 10 min. The resulting PCR products were then purified and cloned into the pMD19-T vector and sequenced commercially (Wuhan Genecreate Biotechnology Co. Ltd., China). The sequence data obtained were analyzed using the DNAMAN software package (ver.8.0. Lynnon Biosolf, California, USA), with the predicted amino acid sequences being used to create the multiple sequence alignments that were utilized to identify amino acid differences between the sequences of the various Sdh subunits from the three resistant mutants and their wild-type parental isolates, as detailed in a previous study [13].

### 2.4. Relative Expression of Four FgSdh Genes in Pydiflumetofen-Resistant Mutants of F. graminearum

Total RNA was extracted from fresh mycelial samples cultured in both the absence and presence of pydiflumetofen (0.1 μg/mL) using a fungal RNA kit (Omega bio-tek, Switzerland) following the protocol of the manufacturer. The first-strand cDNA was then synthesized using the PrimeScript RT reagent kit (TaKaRa, Kusatsu, Japan), with the resulting cDNA being used as a template for the qPCR amplification of partial sequences (approximately 200 bp in length) of the *FgSdhA*, *FgSdhB*, *FgSdhC*, and *FgSdhD* genes with the RT-FgSdhA-F/RT-FgSdhA-R, RT-FgSdhB-F/RT-FgSdhB-R, RT-FgSdhC-F/RT-FgSdhC-R, and RT-FgSdhD-F/RT-FgSdhD-R primer sets, respectively (Table S2). The real-time PCR itself was performed using reaction mixtures containing SYBR Green I fluorescent dye and processed using the QuantStudio 6 Flex PCR detection system (ThermoFisher, Waltham, MA, USA) with the following program: an initial denaturation at 95 °C for 10 s, followed by 40 cycles of 95 °C for 5 s, 60 °C for 34 s, and dissociation at 95 °C for 15 s, 60 °C for 60 s, and 95 °C for 15 s. The relative expression of each gene was then determined following the protocol of a previous study [13,14] using actin as the reference gene with the RT-actin-F/RT-actin-R primer set (Table S2). Each isolate combination was represented by three biological samples, with the resulting values being used to calculate the mean expression and standard error (SE).

### 2.5. Cross-Resistance between Pydiflumetofen and Other Commonly Used Fungicides

The mycelial growth assay used to assess the growth of the pydiflumetofen-resistant mutants was also used to investigate any potential cross-resistance between pydiflumetofen and other commonly used fungicides. The assay was conducted using PDA medium amended with a range of active ingredient concentrations: 0, 0.0005, 0.0015, 0.0045, 0.0135, 0.0405, 0.1215, 0.3645, 1.0935, and 3.2805 μg/mL for tebuconazole, fludioxonil, prochloraz, fluazinam, pyraclostrobin, and difenoconazole as well as pydiflumetofen when assessing the wild-type parental isolates and 0, 1.5625, 3.125, 6.25, 12.5, 25, 50, and 100 μg/mL for carbendazim as well as pydiflumetofen with the pydiflumetofen-resistant mutants. Each treatment/isolate combination was represented by three separate plates, and the entire experiment was performed twice.

### 2.6. Statistical Analysis

The data collected in the mycelial growth, sporulation, and pathogenicity experiments as well as the results from gene expression analysis were subjected to analysis of variance (ANOVA) using SPSS software (ver. 17.0; SPSS Inc., Chicago, IL, USA), with significant differences between treatments being determined using Fisher’s least significant difference test (*p* ≤ 0.05).

## 3. Results

### 3.1. Mycelial Growth and Sporulation of Three Pydiflumetofen-Resistant Mutants of F. graminearum

**Figure 1:**
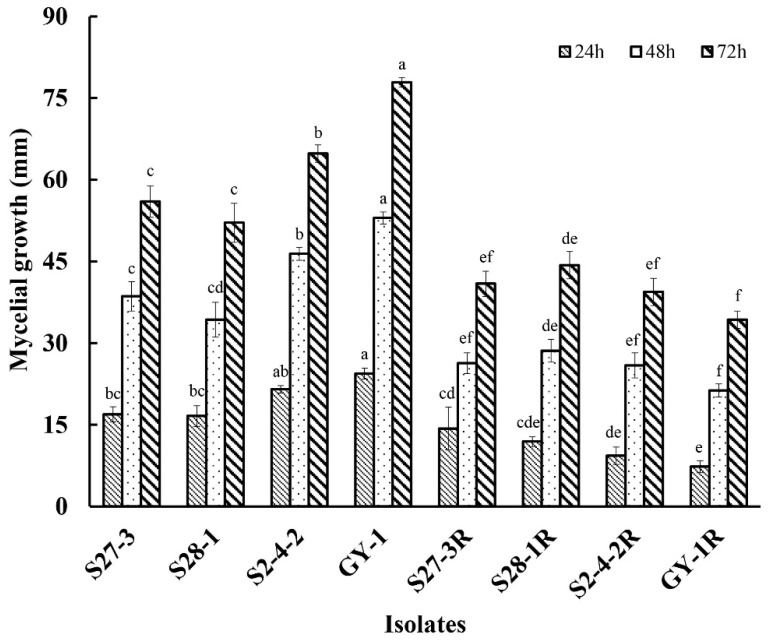
Mycelial growth of three pydiflumetofen-resistant mutants of *F. graminearum.* The average colony diameter of three resistant mutants (R) and their wild-type parental isolates was measured after 24-, 48-, and 72-h incubation on PDA at 24 °C. Data are the means of eight replicates ± standard error (SE). Different lowercase letters above the columns indicate significant differences (*p* ≤ 0.05) after 24, 48, and 72 h incubation according to Fisher’s least significant difference test (*p* ≤ 0.05).

**Figure 2:**
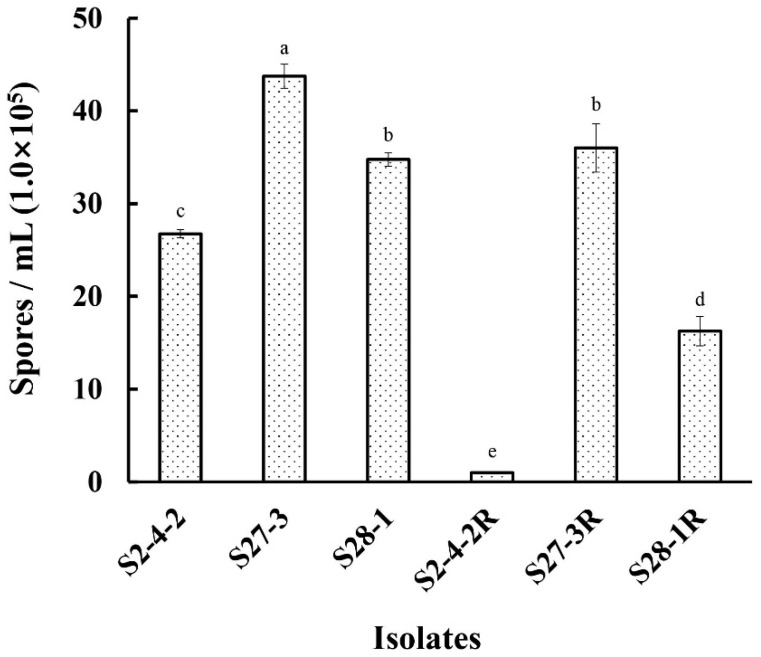
Sporulation of three pydiflumetofen mutants of *F. graminearum.* The sporulation of the resistant mutants (R) and their wild-type parental isolates was assessed after 3 day’s incubation in MBB at 24 °C with shaking (130 rpm). Data are the means of three replicates ± standard error (SE). Different lowercase letters above the columns indicate significant differences (*p* ≤ 0.05) according to Fisher’s least significant difference test (*p* ≤ 0.05).

Although the growth rate of the three pydiflumetofen-resistant mutants (S2-4-2R, S27-3R, and S28-2R) was slightly depressed in the early stages of growth (at 24 h and 48 h), no significant differences (*p* < 0.05) were detected in the diameter of the colonies formed on PDA at 72 h (Figure 1 and Figure S1), which infers that pydiflumetofen resistance had little effect on mycelial growth. However, all of the pydiflumetofen-resistant mutants were found to have significantly reduced levels of sporulation (*p* < 0.05) in comparison to their parental isolates (Figure 2), with the sporulation of one (S28-2R) being reduced by 50% and another (S2-4-2R) to almost zero, which indicates that there is some fitness cost associated with pydiflumetofen resistance in *F. graminearum*.

### 3.2. Pathogenicity of Pydiflumetofen-Resistant Mutants of F. graminearum on Wheat Spikelets

**Figure 3:**
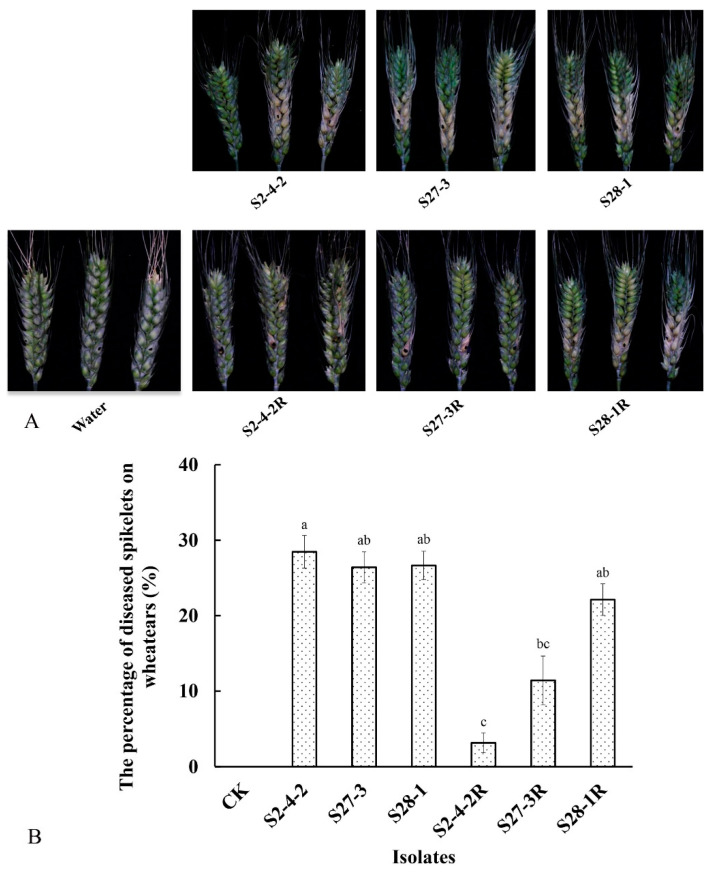
Pathogenicity of three pydiflumetofen-resistant mutants of *F. graminearum*. The top panels (**A**) show the lesions produced by both the resistant mutants (R) and their parental wild-type isolates on wheat spikelets at 14 dpi, while the graph below (**B**) shows the percentage of diseased grains in the spikelets. Data are the means of three replicates ± standard error (SE). Different lowercase letters above the columns indicate significant differences (*p* ≤ 0.05) according to Fisher’s least significant difference test (*p* ≤ 0.05).

All of the pydiflumetofen-resistant mutants in the current study were found to have reduced pathogenicity compared to their wild-type parental isolates (Figure 3). However, there was a high degree of variation between the three mutants, with the difference in one mutant, S2-4-2R, not being significant, while the other two had significantly reduced pathogenicity (*p* < 0.05), resulting in 40% less disease in S27-3R and 80% less in S2-4-2R (Figure 3).

### 3.3. Sequence Analysis of Four FgSdh Genes in Pydiflumetofen-Resistant Mutants of F. graminearum

Numerous point mutations (Figure S2) that resulted in amino acid changes to the predicted sequences of the FgSdh subunits of the pydiflumetofen-resistant mutants were identified by comparing the *FgSdh* sequences from the mutants with those of their parental isolates, which, incidentally, were the same as the corresponding sequences of the *F. graminearum* wild-type species (PH-1) in the *GenBank* database. Interestingly many identical amino acid changes were found to occur in multiple subunits of all three mutants (S2-4-2R, S27-3R, and GY-1R). For example, all of the mutants shared the Y182F and S31F substitutions in their predicted FgSdhA and FgSdhD sequences, respectively (Table 2). Similarly, all of the mutants shared three substitutions (H53Q, C90S, and A94V) in their FgSdhC sequences, while two mutants (S27-3R and S2-4-2R) were found to have an identical change (E39G) in their FgSdhB sequence (Table 2). In addition to these shared amino acid changes, some of the mutants exhibited further substitutions. For example, GY-1R had three single-amino-acid substitutions (H101R, A185V, and W180K) in its FgSdhA, FgSdhC, and FgSdhD subunits respectively, while S2-4-2R had just one further change (A185L) in its FgSdhC sequence, which, interestingly, occurred at the same location as the A185V substitution in the FgSdhC sequence of GY-1R (Table 2). Taken together, these results paint a rather complicated picture of amino acid changes associated with pydiflumetofen resistance in the *F. graminearum* mutants investigated in the current study.

### 3.4. Relative Expression of Four FgSdh Genes in Pydiflumetofen-Resistant Mutants of F. graminearum

**Figure 4:**
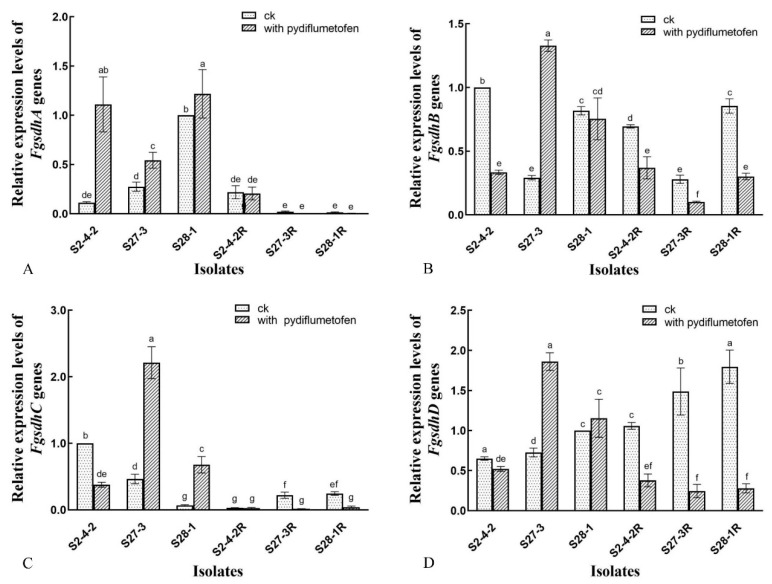
(**A**–**D**) Relative expression of four *FgSdh* genes in pydiflumetofen-resistant mutants of *F. graminearum.* The relative expression of the *FgSdh* genes in the resistant mutants (R) and wild-type parental isolates was compared in both the absence and presence of pydiflumetofen (0.1 μg/mL), using actin as the reference gene. Data are the means of three replicates ± standard error (SE). Different lowercase letters above the columns indicate significant differences (*p* ≤ 0.05) in the absence and presence of pydiflumetofen (0.1 μg/mL) according to Fisher’s least significant difference test (*p* ≤ 0.05).

The results of the qPCR experiments (Figure 4) were a little difficult to interpret, as the genetic background of the parental isolates (S2-4-2, S27-3, and GY-1) seems to influence gene expression even before considering any altered expression that might occur in the resistant mutants. For example, the expression of *FgSdhA* followed the same general pattern in all of the parental isolates, with pydiflumetofen treatment resulting in a significant increase in the expression in all three isolates, while quite a different pattern of expression was observed for *FgSdhB* in response to the fungicide, with expression being dramatically down-regulated in S2-4-2, dramatically up-regulated in S27-3, and little changed in GY-1. However, some general trends could be observed in the pydiflumetofen-resistant mutants. For example, all of the mutants exhibited remarkably low expression of both their *FgSdhA* and *FgSdhC* genes, which appeared to be down-regulated even further by the presence of pydiflumetofen in the case of *FgSdhC*. Similarly, all of the mutants exhibited a shared pattern of expression with regard to their *FgSdhB* and *FgSdhD* genes, which resulted in high levels of expression in the absence of pydiflumetofen that were significantly higher (*p* < 0.05) than the parental isolates in the case of *FgSdhD* but also significant down-regulation (*p* < 0.05) in the presence of the fungicide. Indeed, the mutants seemed to exhibit more consistent patterns of gene expression than the wild-type parental isolates, indicating that the shared amino acid changes detailed in the preceding section might have had an effect on gene expression that superseded the divergence in expression resulting from the genetic background the mutants inherited from their parental isolates.

### 3.5. Cross-Resistance between Pydiflumetofen and Other Commonly Used Fungicides

Although the current study found that pydiflumetofen was a highly effective fungicide against wild-type isolates of *F. graminearum* (S2-4-2, S27-3, and S28-2), with EC_50_ values of 0.0235, 0.0486, and 0.2354 μg/mL, respectively, its efficacy was greatly reduced in the resistant mutants (S2-4-2R, S27-3R, and S28-2R), which had values of 25.10, 28.57, and 19.22 μg/mL (Table 1). However, despite this dramatic change to pydiflumetofen sensitivity, no evidence of cross-resistance was found between pydiflumetofen and the other fungicides tested, which included tebuconazole, fludioxonil, prochloraz, fluazinam, carbendazim, pyraclostrobin, and difenoconazole (Table 1).

## 4. Discussion

The succinate dehydrogenase inhibitor (SDHI) group of fungicides is highly effective against a broad range of fungi and widely used to control many plant diseases [10,15,16,17]. However, since SDHI fungicides have a single target site, many plant pathogens have already developed resistance. Indeed, there is growing evidence that SDHI resistance is primarily caused by amino acid mutations in the vicinity of the ubiquinone-binding pocket, especially in the SdhB, SdhC, and SdhD subunits [12,18,19]. Such mutations have been documented in many plant pathogens including *Mycosphaerella graminicola* [20], *Alternaria alternata* and *Alternaria solani* [19,21], *Zymoseptoria tritici* [21], *Blumeriella jaapii* [22], and *Clarireedia jacksonii* [23] although different pathogens may exhibit different resistance mechanisms to the same fungicide.

The new-generation SDHI fungicide, pydiflumetofen, has been registered in China since 2020, primarily for the control of FHB in wheat (http://www.chinapesticide.org.cn/hysj/index.jhtml, accessed on 30 October 2022). However, to date, there has been little investigation into potential resistance mechanisms in *F. graminearum*. The current study found that although three laboratory mutants of *F. graminearum* were highly resistant to pydiflumetofen, this resistance was accompanied by a certain cost to fitness, including significantly reduced sporulation and reduced pathogenicity in wheat spikelets even though mycelial growth was little affected (Figure 1, Figure 2 and Figure 3). These results are in contrast to a previous study of *F. asiaticum*, which found that pydiflumetofen-resistant mutants were completely unimpaired with regard to mycelial growth, sporulation, and virulence [12], and could suggest a lower risk associated with SDHI resistance in *F. graminearum* compared to *F. asiaticum*. Meanwhile, current studies provide further evidence of a fitness cost associated with pydiflumetofen resistance in *F. graminearum* but also indicate that there can be some degree of variation in different mutants, which could possibly be related to the molecular biology underlying the specific resistance mechanism of each mutant.

Many previous studies have documented amino acid substitutions associated with SDHI resistance in plant pathogenic fungi, particularly the conserved histidine residues in the ubiquinone binding site of the Sdh complex [24], for example, the H272L/R/Y mutations in the SdhB subunit of *B. cinerea* [10,25,26,27], the H132R mutation in the SdhD subunit of both *B. cinerea* and *Sclerotinia sclerotiorum* [28,29], and the H277Y/R/L, H134R, and H133R/P/T mutations in the SdhB, SdhC, and SdhD subunits, respectively, of *A. alternata* [30,31]. Single-amino-acid mutations have also been noted in previous studies of pydiflumetofen resistance in *Fusarium* species, including the A83V or R86H/C substitutions in the SdhC subunit of *F. graminearum* [8] as well as the H248Y and A64V or R67K substitutions in the SdhB and SdhC subunits, respectively, of *F. asiaticum* [12]. In contrast, the current study identified a collection of amino acid substitution in multiple subunits such that all three *F. graminearum* mutants exhibited five identical changes, including Y182F in the FgSdhA subunit; H53Q, C90S, and A94V in FgSdhB; and S31F in FgSdhC (Table 2). In addition, several mutants also exhibited further mutations, including H101R in FgSdhA, E39G in FgSDhB, W180K in FgSdhD, and A185V/L in FgSdhC, the latter of which resulted in two mutants having amino acid changes in all four of their Sdh subunits. To the best of our knowledge, this is the first report of these amino acid changes being associated with pydiflumetofen resistance in *Fusarium* as well as the first report of changes in multiple subunits or in the SdhA subunit, with the majority of changes linked with SDHI resistance generally occurring in subunits B, C, and D [24]. In addition, the current study found that the expression of all four FgSdh subunits was significantly altered (*p* < 0.05) compared to that of the parental wild-type isolates, with FgSdhA and FgSdhC in particular showing remarkably low levels of expression (Figure 4). However, it is unclear whether this altered expression contributed to the observed pydiflumetofen resistance or was merely a consequence of the primary resistance mechanism. Further studies, including site-directed mutagenesis for each amino acid change, both in isolation and in combination, are therefore required to evaluate the contribution of each to the resistance mechanism occurring in the *F. graminearum* mutants of the current study.

Although the multiple mutations and fitness cost reported in the current study suggest that the risk of pydiflumetofen resistance in *F. graminearum* is not particularly high, previous studies have found that the risk of resistance to SDHI fungicides in plant pathogenic fungi is medium to high [11,32,33]. Indeed, recent reports have shown that the in vitro selection of pydiflumetofen-resistant mutants can occur spontaneously in both *F. graminearum* and *F. asiaticum*, without the need for complex mutagenic procedures [8,12]. Consequently, great care should be taken in developing management strategies that both provide effective control of FHB in the field but also limit the potential for fungicide resistance to emerge. It was therefore extremely encouraging that the current study found no evidence of cross-resistance between pydiflumetofen and any of the other fungicides assessed, including tebuconazole, fludioxonil, prochloraz, fluazinam, carbendazim, pyraclostrobin, and difenoconazole, which indicates that the use of pydiflumetofen either in rotation or combination with these fungicides could provide ongoing control of FHB and ensure high and stable wheat yields in China for many years to come.

## Figures and Tables

**Table 1:** Cross-resistance between pydiflumetofen and other commonly used fungicides.

Fungicides	Pydiflumetofen-Sensitive Isolates (EC_50,_ μg/mL)			Pydiflumetofen-Resistant Mutants (EC_50,_ μg/mL)		
	S2-4-2	S27-3	S28-1	S2-4-2R	S27-3R	S28-1R
Pydiflumetofen	0.024	0.049	0.235	25.100	28.570	19.220
Tebuconazole	0.119	0.083	0.074	0.426	0.052	0.632
Fludioxonil	0.003	0.003	0.005	0.010	0.012	0.014
Prochloraz	0.003	0.002	0.004	0.010	0.011	0.013
Fluazinam	0.011	0.009	0.012	0.011	0.009	0.015
Carbendazim	0.429	0.552	0.636	0.486	0.265	0.612
Pyraclostrobine	0.041	0.045	0.126	0.022	0.021	0.046
Difenoconazole	0.103	0.286	0.645	0.190	0.008	0.641

**Table 2:** Point mutations in *Sdh* genes associated with pydiflumetofen resistance in *F. graminearum* and *F. asiaticum*.

Genes	Mutants	Nucleotide Mutations	Amino Acid Changes	Species/Reference
*SdhA*	S27-3R	A833T, C834T	Y182F	*F. graminearum*/Current study
*SdhA*	S2-4-2R	A833T, C834T	Y182F	*F. graminearum*/Current study
*SdhA*	GY-1R	A539G, A833T, C834T	H101R, Y182F	*F. graminearum*/Current study
*SdhB*	S27-3R	A116G	E39G	*F. graminearum*/Current study
*SdhB*	S2-4-2R	A116G	E39G	*F. graminearum*/Current study
*SdhB*	GY-1R	/	/	*F. graminearum*/Current study
*SdhB*	/	*/*	H248Y	*F. asiaticum*/Chen et al., 2021
*SdhC*	S27-3R	C270G, G380C, C392T	H53Q, C90S, A94V	*F. graminearum*/Current study
*SdhC*	S2-4-2R	C270G, G380C, C392T, G664C, C665T	H53Q, C90S, A94V, A185L	*F. graminearum*/Current study
*SdhC*	GY-1R	C270G, G380C, C392T, C665T	H53Q, C90S, A94V, A185V	*F. graminearum*/Current study
*SdhC*	*/*	*/*	A83V	*F. graminearum*/Sun et al., 2020
*SdhC*	*/*	*/*	R86H/C	*F. graminearum*/Sun et al., 2020
*SdhC*	*/*	*/*	A64V	*F. asiaticum*/Chen et al., 2020
*SdhC*	*/*	*/*	R67K	*F. asiaticum*/Chen et al., 2020
*SdhD*	S27-3R	C92T	S31F	*F. graminearum*/Current study
*SdhD*	S2-4-2R	C92T	S31F	*F. graminearum*/Current study
*SdhD*	GY-1R	C92T, G678A	S31F, W180K	*F. graminearum*/Current study

/: Indicates no data available.

## Data Availability

Not applicable.

## Supplementary Materials

The following are available online at: https://www.mdpi.com/article/10.3390/jof9010062/s1, Figure S1: Mycelial growth of three pydiflumetofen-resistant mutants of *F. graminearum* and their sensitive parental isolates; Figure S2: Multiple sequence of the *FgSdhA, FgSdhB*, and *FgSdhC* genes in pydiflumetofen-resistant mutants of *F. graminearum* and their sensitive parental isolates; Table S1: Fungicides used in the current study; Table S2: Primers used in the current study.
