# Supplementary material for: Mechanism of Pydiflumetofen Resistance in Fusarium graminearum in China
Source: J Fungi (Basel). 2022 Dec 30;9(1):62. doi: 10.3390/jof9010062 (PMC9866472; doi:10.3390/jof9010062)
Supplement: Supplementary file 1 [file jof-09-00062-s001.zip › jof-2033457-supplementary.pdf]

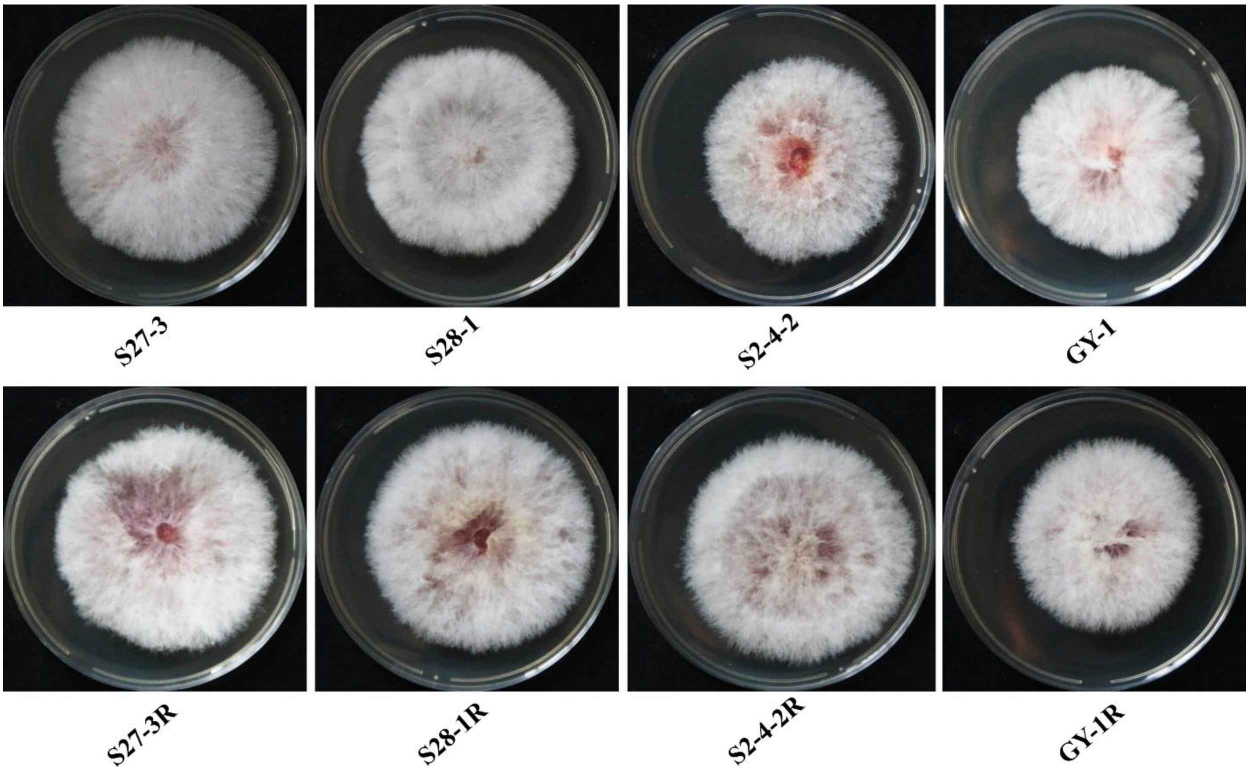

Figure S1. Mycelial growth of three pydiflumetofen-resistant mutants of *F. graminearum* and their sensitive parental isolates.

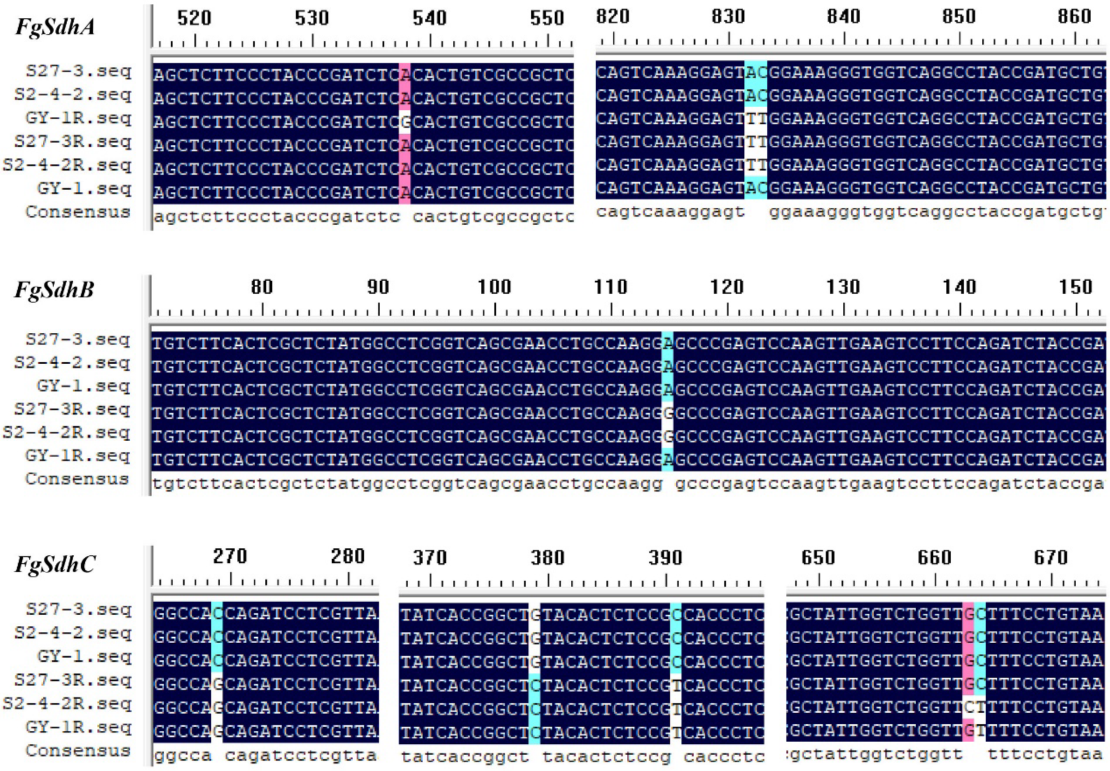

Figure S2. Multiple sequence of the *FgSdhA*, *FgSdhB*, and *FgSdhC* genes in pydiflumetofen-resistant mutants of *F. graminearum* and their sensitive parental isolates.

Table S1. Fungicides used in the current study.

Fungicides				Source
Common names	Fungicide groups	Mode of action target	FRAC[N] code	
98.0% Pydiflumetofen	Respiration	SDHI (Succinate dehydrogenase inhibitors)	7	Syngenta Investment Co., Ltd., China
96.2% Tebuconazole	DMI（14α-demethylase inhibitor）	Sterol biosynthesis in membranes	3	Sheyang Huanghai Pesticide Chemical Co. Ltd.
96.0% Fludioxonil	Phenylpyrrole	MAP/Histidine-Kinase in osmotic signal transduction	12	Hubei Jianyuan Chemical Co. Ltd.
95% Fluazinam	Respiration	Uncouplers of oxidative phosphorylation	29	Zhejiang Heben Pesticide & Chemicals Co. Ltd.
97.5% Pyraclostrobin	Respiration	Complex III: cytochrome bc1 (ubiquinol oxidase) at Qo site (cyp b gene)	11	Kangbaotai Fine-Chemical Co. Ltd.
95.0% difenoconazole	Sterol biosynthesis in membranes	DMI-fungicides (Demethylation inhibitions) (SBI: Class I) Azole	3	Hubei Jianyuan Chemical Co. Ltd.
98.1% Carbendazim	Methyl benzimidazole carbamate	ß-tubulin assembly in mitosis	1	Haili Guixi Chemical Co. Ltd.

[N]FRAC, Fungicide Resistance Action Committee.

Table S2. Primers used in the current study.

Primers	Sequences (5′ to 3′)	Application
FgSdhA F	AACCATATCCAACAAAAGTCCTACTC	Target genes amplification
FgSdhA R	AACTCTTACTACAAAGGAGACGCTG	
FgSdhB F	CTAACGGAGGGGATTGTACGGCATTT	
FgSdhB R	TAACCCTTCAGATCGCTTATGCCTCC	
FgSdhC F	TACCAGCCCTCTTTGGAGTAACGACG	
FgSdhC R	CTCGTCCAGACTCCTTTCCCTATATC	
FgSdhD F	AACTCTAACTACTCCAAAAAGCCCT	
FgSdhD R	ATACAGATGCCCGCAGTCATAAACCT	
RT-actin-F	GTCCACCTTCCAGCAAATGT	Target genes expression
RT-actin-R	CCCAAAGCTTAGCGTCTGTC	
RT-FgSdhA-F	AGGCTCCCGCTTCCATTAT	
RT-FgSdhA-R	TTCCGTACTCCTTTGACTGACC	
RT-FgSdhB-F	GCCCATCTTACTGGTGGAACT	
RT-FgSdhB-R	GTGGCAACGGTACAGACTCATT	
RT-FgSdhC-F	GCTCTGGCTGCTGGAATCTC	
RT-FgSdhC-R	GCTACCGAACCATGTCTGCTC	
RT-FgSdhD-F	TTGACCGTCGCTCCCTTTG	
RT-FgSdhD-R	TCTCGAACTCGTACAGTCCCAC	
